# Information technology-supported integrated health service for older adults in long-term care settings

**DOI:** 10.1186/s12916-024-03427-7

**Published:** 2024-05-29

**Authors:** Jung-Yeon Choi, Hongsoo Kim, Seungyeon Chun, Young-il Jung, Sooyoung Yoo, In-Hwan Oh, Gi-Soo Kim, Jin Young Ko, Jae-Young Lim, Minho Lee, Jongseon Lee, Kwang-il Kim

**Affiliations:** 1 Departments of Internal Medicine, Seoul National University Bundang Hospital, Seongnam, Republic of Korea; 2 Department of Public Health Sciences, Graduate School of Public Health, Seoul National University, Seoul, Republic of Korea; 3 Institute of Health and Environment, Seoul National University, Seoul, Republic of Korea; 4 Institute of Aging, Seoul National University, Seoul, Republic of Korea; 5 Department of Environmental Health, Korea National Open University, Seoul, Republic of Korea; 6 Healthcare ICT Research Center, Seoul National University Bundang Hospital, Seongnam, Republic of Korea; 7 Department of Preventive Medicine, Kyung Hee University, Seoul, Republic of Korea; 8 Department of Industrial Engineering, Ulsan National Institute of Science and Technology, Ulsan, Republic of Korea; 9 Department of Rehabilitation Medicine, Seoul National University Bundang Hospital, Seongnam, Republic of Korea; 10 Department of Rehabilitation Medicine, Seoul National University College of Medicine, Seoul, Republic of Korea; 11 Healthcare Convergence R&D Center, ezCaretech Co. Ltd, Seoul, Republic of Korea; 12 Healthcare Convergence R&D Center, Healthconnect Co. Ltd, Seoul, Republic of Korea; 13 Departments of Internal Medicine, Seoul National University College of Medicine, 82 Gumi-ro, 173 Beon-gil, Bundang-gu, Seongnam-si, Kyeongi-do 13620 Republic of Korea

**Keywords:** Health services for the aged, Information technology, Long-term care

## Abstract

**Background:**

To examine the effectiveness and safety of a data sharing and comprehensive management platform for institutionalized older patients.

**Methods:**

We applied information technology-supported integrated health service platform to patients who live at long-term care hospitals (LTCHs) and nursing homes (NHs) with cluster randomized controlled study. We enrolled 555 patients aged 65 or older (461 from 7 LTCHs, 94 from 5 NHs). For the intervention group, a tablet-based platform comprising comprehensive geriatric assessment, disease management, potentially inappropriate medication (PIM) management, rehabilitation program, and screening for adverse events and warning alarms were provided for physicians or nurses. The control group was managed with usual care. Co-primary outcomes were (1) control rate of hypertension and diabetes, (2) medication adjustment (PIM prescription rate, proportion of polypharmacy), and (3) combination of potential quality-of-care problems (composite quality indicator) from the interRAI assessment system which assessed after 3-month of intervention.

**Results:**

We screened 1119 patients and included 555 patients (control; 289, intervention; 266) for analysis. Patients allocated to the intervention group had better cognitive function and took less medications and PIMs at baseline. The diabetes control rate (OR = 2.61, 95% CI 1.37–4.99, *p* = 0.0035), discontinuation of PIM (OR = 4.65, 95% CI 2.41–8.97, *p* < 0.0001), reduction of medication in patients with polypharmacy (OR = 1.98, 95% CI 1.24–3.16, *p* = 0.0042), and number of PIMs use (*ꞵ* =  − 0.27, *p* < 0.0001) improved significantly in the intervention group. There was no significant difference in hypertension control rate (OR = 0.54, 95% CI 0.20–1.43, *p* = 0.2129), proportion of polypharmacy (OR = 1.40, 95% CI 0.75–2.60, *p* = 0.2863), and improvement of composite quality indicators (*ꞵ* = 0.03, *p* = 0.2094). For secondary outcomes, cognitive and motor function, quality of life, and unplanned hospitalization were not different significantly between groups.

**Conclusions:**

The information technology-supported integrated health service effectively reduced PIM use and controlled diabetes among older patients in LTCH or NH without functional decline or increase of healthcare utilization.

**Trial registration:**

Clinical Research Information Service, KCT0004360. Registered on 21 October 2019.

**Supplementary Information:**

The online version contains supplementary material available at 10.1186/s12916-024-03427-7.

## Background

As the population ages, the number of older adults who require institutional long-term care (LTC) in settings such as long-term care hospitals (LTCHs) or nursing homes (NHs) is increasing worldwide [1]. However, the needs for better access and high-quality services is growing [2]. Although older patients’ complexity of care increases due to multimorbidity and disability, LTCHs and NHs are frequently understaffed. The coronavirus disease 2019 (COVID-19) pandemic has had a particularly negative impact on older patients staying in LTC settings [3]. Isolation and limited accessibility of specialized care have proven to be a great burden in managing such older patients [4].

Lack of medical information sharing causes misunderstanding for the goals of the patient’s medical treatment and care, which could result in adverse drug events and rehospitalization [5, 6]. The shortage of specialists in caring for older patients is another problem worldwide [7]. For the optimal care of older patients, a well-organized geriatric team comprising a geriatrician, nurse, dietician, pharmacist, and physical therapist is required to improve patient and healthcare system outcomes [8]. In LTC settings, it is difficult to maintain a specialist team to care for older patients; however, specialized geriatric care can be accessed with the help of new technology. Advances in information and communication technology (ICT) have facilitated healthcare professionals in maintaining communication with their patients and making appropriate medical decisions [9]. Furthermore, telemedicine positively affected the management of diabetes and hypertension, the most common chronic diseases among the older population, with consultation and tele-monitoring [10].

Several programs have been developed to promote interoperability between acute hospitals and LTCHs or NHs. However, there are significant shortcomings in the completeness, timeliness, and usability of information sharing between facilities [6]. There are also unsolved problems, such as standardized formats for information sharing, establishment of standard workflow, and development of data sharing platforms  .

To overcome these challenges, we developed a new service model and system using an ICT called Health-RESPECT (integrated caRE Systems for elderly PatiEnts using iCT) [11, 12]. The Health-RESPECT platform includes comprehensive geriatric assessment (CGA), a standardized management algorithm for chronic diseases, individualized rehabilitation protocol, medication screening and management, and consultation services with acute care hospitals. Furthermore, we established a system for data collection to share the vital data of older patients staying in LTCHs or NHs, such as blood pressure, pulse rate, blood glucose level, or other blood tests. Medication prescription details were also collected using the national referral service program [13, 14].

This study assessed whether interventions using Health-RESPECT can improve the clinical outcomes of older patients in Korean LTC settings. Institutions providing LTC in Korea are divided into LTCHs and NHs. The former is covered by the National Health Insurance Scheme and provides long-term hospital beds for treatment, while the latter is covered by LTC insurance and mainly provides assistive welfare services (nursing, caregiving, etc.). However, despite these institutional differences, the clinical characteristics of patients were reported to be similar [15]. Our principal hypothesis was that an intervention using the Health-RESPECT platform would show significant benefits in chronic disease management, medication management, and physical and cognitive functional status.

## Methods

### Study design overview

The Health-RESPECT study is a pragmatic, multicenter, cluster randomized controlled trial. Cluster randomization was performed using the institutional identification number. The LTCH and NH were recruited and randomly allocated to the intervention or control group. The LTCH and NH located at Seoul or Gyeonggi-do were recruited and randomly allocated to the intervention or control group. We started recruiting participants in September 2019. The details of the Health-RESPECT study were published in a previous protocol paper [12]. The present study was registered with the Clinical Research Information Service (CRIS, https://cris.nih.go.kr/, trial registration number: KCT0004360) (initial submission date: September 4, 2019). The study protocol was reviewed by the Seoul National University Bundang Hospital institutional review board (IRB No. B-1908/556–304). The study design adhered to the principles of the Declaration of Helsinki.

### Participants

Patients aged over 65 years, who were admitted to LTCHs or NHs for the management of multiple medical conditions such as dementia or functional disability, who had stayed at the facility for at least two weeks before enrollment in the study, and who had at least one chronic disease (hypertension, diabetes, heart failure, etc.) were included in the study. We excluded patients whose life expectancy was less than 3 months, as assessed by physicians at the institution. Patients who were scheduled for discharge within 3 months or refused to participate in this study were also excluded. Written informed consent was obtained from study participants with intact cognition, or from a health care proxy on behalf of participants with impaired cognition.

### Health-RESPECT platform and data acquisition

Data of vital signs (blood pressure, heart rate, and body temperature), laboratory findings, and prescribed medications are collected and updated from the electronic medical records (EMR) of the LTCH every month by nationwide health information exchange (HIE) system [16–18] (Additional file 1: Fig S1). Through the collected data, an individualized management plan for chronic disease, medication, and rehabilitation is provided according to the algorithm within the platform. Health RESPECT program provides management goals and specific comments based on a determined decision support algorithm, considering the patients’ frailty and disease status. For the Health-RESPECT clinical study, we established a new HIE system for the participating LTC institutions. We ensured that all vital signs were uploaded from the participating sites to our Health-RESPECT platform.

### Measurement

Frailty status was evaluated at enrollment using the Korean version of the FRAIL (K-FRAIL) scale [19, 20]. Patients with scores ≥ 3 were classified as frail, those with scores of 1 to 2 were pre-frail, and 0 were robust. Cognitive and physical function were evaluated using the Korean version of the Mini-Mental State Examination (MMSE-K) and functional ambulation category (FAC), respectively. Activities of daily living (ADL) were evaluated using the ADL Hierarchy Scale, which tests four items (personal hygiene, toilet use, locomotion, and eating) and ranges from 0 to 6, with higher scores indicating increased ADL dependency [21].

Treatment targets for hypertension and diabetes differ according to frailty status. The target blood pressure for hypertension was 140/90 mm Hg in the robust and prefrail groups and 150/90 mm Hg in the frail groups. The target hemoglobin A1c (HbA1c) for diabetes was < 7.5% in robust groups, < 8.0% in prefrail groups, and < 8.5% in frail groups, or for random glucose levels, the target was ≤ 190 mg/dL in robust groups, ≤ 210 mg/dL in prefrail groups and ≤ 230 mg/dL in frail groups [12]. Data on patients’ vital signs, laboratory findings, diagnosis, and prescribed medication were collected from the electronic medical records of the LTC institutions and accumulated in Health-RESPECT. Potentially inappropriate medication (PIM) and polypharmacy were determined based on medications from the prescribed record.

Health-related quality of life (HRQoL) was assessed using the Korean version of the EuroQol instrument (Korean preference weighted EuroQol 5-Dimensions (EQ-5D) [22, 23]. The EQ-5D comprises five dimensions (mobility, self-care, usual activities, pain or discomfort, and anxiety or depression), each of which can be rated at three levels (no problem, some problems, severe problems) [24]. For the EQ-VAS, patients were asked to rate their current health state on a scale of 0 (worst imaginable) to 100 (best imaginable).

The list of PIMs was defined by referring to the Beers Criteria 2019 and considering the medical environment of the LTC institutions in Korea [25]. A detailed list of medications was presented in the protocol paper and Additional file 1: Table S1 [12].

The composite quality-of-care indicator (CQI) computed from the interRAI assessment system (interRAI CQI) measures the effects of intervention by the overall change in participants’ functional status [25]. It comprises a set of individual QIs (inadequate pain management, little or no activity, physical restraint use, etc.). The CQI score was calculated as the sum of 33 individual QIs indicating whether an older adult is at risk for functional decline or needs clinical interventions [26, 27].

### Randomization and intervention

In our study, one university hospital, seven LTCHs, and six NHs were recruited through a medical referral team of a university hospital where they communicate and transfer patients to LTC facilities. University hospital and LTC use HIE systems to exchange medical information. Although there are various EMR companies and systems in Korea, we could only carry out additional development with the two companies with the highest market share in LTC facilities to connect HIE systems with the Health-RESPECT system. So, LTC facilities were recruited when (1) expressed interest in our study when the medical referral team contacted (2) used one of the two major EMR company systems, and (3) agreed to participate in this study through a face-to-face meeting.

To account for the potential impact of institutional factors on the efficacy of the intervention, the age distribution of patients was utilized as a proxy for disease, clinical condition, and functional status. Accordingly, institutions were randomized after the proportion of older adults aged over 75 years was matched. The LTCH and NH were allocated into the intervention and control groups at a 1:1 ratio. Given the introduction of Health-RESPECT as an intervention, the study participants could not be blinded. To maintain anonymity, the assessors and the statistician were blinded to the allocation of facilities until their role was complete.

**Fig. 1:**
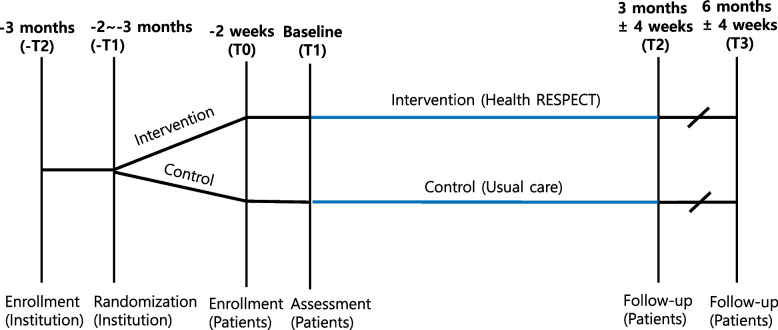
Study timeline for the Health RESPECT study. This figure illustrates the timeline of the Health RESPECT study from initial enrolment to follow-up assessments. Baseline assessments (T1) are conducted at the start, followed by subsequent evaluations at 3 months (T2) and 6 months (T3)

The intervention comprised a CGA; evidence-based management algorithm for hypertension, diabetes, and heart failure (Additional file 1: Fig S2); screening for PIM; tailored cognitive, physical, and swallowing rehabilitation program; and alarm for adverse events. Access to medical information within Health-RESPECT was granted only to medical staff. The CGA of Health-RESPECT consisted of a digitalized “patient evaluation form,” which must be completed and submitted periodically for reimbursement from LTCHs, along with additional assessment from nurses at LTCHs and NHs (ADL, IADL, K-FRAIL, and FAC). Nurses at LTCHs and NHs conducted CGA. The usage of the Health-RESPECT and the measurement method was described in detail in a manual book and distributed to each LTCH and NH, and the research nurse visited the site in person to provide education if needed. The medication management program screened all prescribed medications and provided the number and specified PIM list corresponding to the absolute or potentially inappropriate drug list. Patients randomized to the control group were managed with usual care without any exposure to assessment methods and intervention tools. Fig. 1.

### Outcome and follow-up

Primary and secondary outcomes were evaluated at 3 months after the intervention or observation period by independent assessors trained by the research team. The co-primary outcomes were: (1) control rate of hypertension and diabetes, (2) medication adjustment (PIM prescription rate, the proportion of polypharmacy discontinuing), and (3) combination of potential quality-of-care problems (CQI) from the interRAI Long Term Care Facility (LTCF) assessment system, which is widely used in about 35 countries; the system consists of a psychometrically sound CGA instrument, a manual, and applications including scales and quality indicators [26, 27]. The hypertension and diabetes control rate was determined considering the frailty status [28, 29]. Detailed descriptions of primary and secondary outcomes were attached to the supplementary file (Additional file 1: Table S2).

### Statistical analysis

The sample size was calculated based on the reduction of PIM prescriptions. We expected that the intervention would reduce PIM by 20% as compared to the control group. Assuming a 5% significance, 80% power, and an intra-cluster correlation coefficient of 0.01, we calculated that at least 480 participants were needed. The primary analysis was intention-to-treat (ITT) and the secondary analysis was per-protocol (PP).

Continuous variables were expressed as mean ± standard deviation or median and interquartile range and compared by unpaired Student’s t-test. Categorical variables were expressed as counts and percentages, and the chi-square test was used to compare proportions. We analyzed the difference in outcomes between the intervention and control groups by fitting a linear mixed effects model (LMM) and a generalized linear mixed effects model (GLMM) to account for the correlation between patients hospitalized in the same LTCH or NH and repeated observations of the same patient. Specifically, two random intercepts were introduced, one at the LTC institution level using an identification number (ID) and the other at the patient level. The LTC institution-ID random intercepts capture the variability in the outcome among different LTC institutions. Furthermore, the random effects adjust for the difference in outcomes between LTC institutions at the baseline. Statistical analyses were performed with SAS (version 9.4, SAS Institute, Cary, NC). All statistical analyses were two-tailed, and *p*-values < 0.05 were considered statistically significant.

## Results

**Fig. 2:**
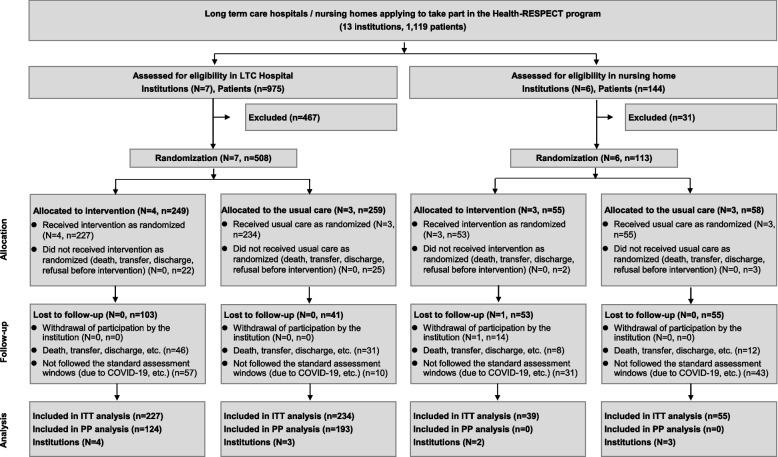
Flow diagram of participants of the Health-RESPECT study. Flow chart describing the number of institutions and participants who were enrolled in the study, who were excluded or dropped out of the study, and included in the analysis

We invited LTCHs (*N* = 7) and NHs (*N* = 6) to participate in this study. A total of 1119 patients were recruited (975 from LTCHs; 144 from NHs). A cluster of 4 LTCHs and 3 NHs were allocated to the intervention group, and 3 LTCHs and 3 NHs were assigned to the control group. After excluding patients who did not receive any intervention using the Health-RESPECT platform, we included 555 patients (control; 289, intervention; 266) for ITT analysis. Baseline assessments were conducted from Sept 2019 to Dec 2020, and primary outcomes were assessed after the 3 months of intervention or observation period. There were 252 patients lost to follow-up (control; 96, intervention; 156). Among them, 141 patients (control; 53, intervention; 88) could not complete the scheduled assessments within the given window time due to the COVID-19 pandemic, and 97 patients (control; 43, intervention; 54) were lost to follow-up due to death/transfer/discharge (Fig. 2).

The demographic characteristics of study participants are presented in Table 1. Compared with the control group, patients allocated to the intervention group had higher BMI (21.4 kg/m^2^ vs 20.7 kg/m^2^, *p* = 0.0396), and took less medications (9.5 vs. 11.4, *p* < 0.0001) and fewer number of PIMs (0.9 vs. 1.4, *p* < 0.0001). However, there was no significant difference in length of stay, prevalence of chronic diseases, ADL hierarchy scale, cognitive performance scale, HRQoL, and frailty status at baseline.

**Table 1:** Comparison of the baseline characteristics between the control and intervention groups

	**Control group** **(*n* = 289)**	**Intervention group** **(*n* = 266)**	***p* value**
Age (year), mean (SD)	81.9 (7.5)	82.5 (6.8)	0.3291
Male sex, *n* (%)	74 (25.6)	63 (23.7)	0.6000
Medicaid, *n* (%)	34 (11.8)	19 (7.1)	0.0642
Length of stay (day), median (IQR)	375 (155, 863)	354 (140, 890)	0.5515
Number of diseases, mean (SD)	1.9 (1.1)	1.7 (1.0)	0.1105
Hypertension, *n* (%)	227 (78.6)	223 (83.8)	0.1121
Diabetes mellitus, *n* (%)	139 (51.9)	112 (42.1)	0.1565
CHF, *n* (%)	26 (9.0)	25 (9.4)	0.8699
BMI (kg/m^2^), mean (SD)	20.7 (3.8)	21.4 (4.1)	***0.0396***
ADL hierarchy scale, mean (SD)	3.7 (1.8)	3.9 (1.8)	0.1572
Cognitive performance scale, mean (SD)	2.8 (1.7)	2.6 (1.5)	0.1812
FAC, mean (SD)	0.8 (1.3)	0.9 (1.4)	0.4022
EQ-5D, mean (SD)	0.55 (0.26)	0.51 (0.27)	0.0731
Frailty, *n* (%)	120 (41.5)	103 (39.0)	0.5483
CQI, mean (SD)	3.7 (2.1)	3.6 (2.2)	0.8914
Number of medications, mean (SD)	11.4 (4.9)	9.5 (4.1)	** < *0.0001***
Number of PIM, mean (SD)	1.3 (1.1)	0.9 (1.0)	** < *0.0001***

*ADL* activities of daily living, *BMI* body mass index, *CHF* congestive heart failure, *CQI* composite quality indicators, *FAC* functional ambulation categories, *EQ-5D* EuroQol 5-Dimensions utility weight, *PIM* potentially inappropriate medication

Data are presented as number (%), mean (SD), or median (IQR)

**Fig. 3:**
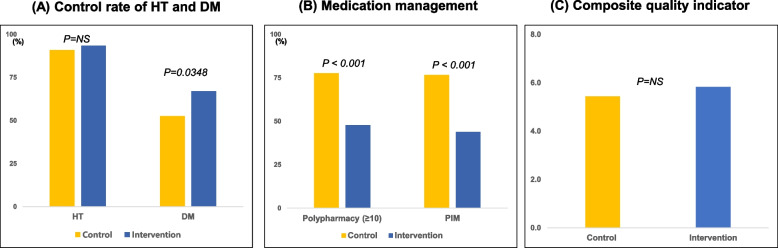
Outcomes of intervention on Health-RESPECT. **A** Control rates of hypertension (HT) and diabetes mellitus (DM) comparing control and intervention groups. **B** Medication management focusing on polypharmacy (≥ 10 medications) and potentially inappropriate medications (PIM). **C** Composite quality indicator scores for the control and intervention groups showed no significant differences. NS; non-significant

With the use of the Health-RESPECT platform, the diabetes control rate improved significantly in the intervention group. However, there was no significant change in the hypertension control rate (Fig. 3A). The number of patients taking multiple medications (polypharmacy defined as taking more than 10 medications) and PIM decreased significantly in the intervention group (Fig. 3B). A greater proportion of patients discontinued more than one inappropriate medication in the intervention group. However, there was no significant difference in CQI between intervention and control groups (Fig. 3C). Analysis of primary outcomes using the mixed effects model showed that diabetes control was significantly improved with the Health-RESPECT intervention (adjusted OR = 2.61, 95% CI: 1.37–4.99, *p* = 0.0035).

Furthermore, the number of PIM use significantly reduced in the intervention group (*ꞵ* =  − 0.27, *p* < 0.0001). In the intervention group, there was a significant increase in the number of patients who discontinued one or more PIMs (adjusted OR = 4.65, 95% CI: 2.41–8.97, *p* < 0.0001) as well as a significant increase in the number of patients who reduced their medication by more than one (adjusted OR = 1.98, 95% CI: 1.24–3.16, *p* = 0.0042) (Table 2). The number of heart failure patients was too small, so the analysis details were not presented in Table 2. We also presented the primary outcome with the per protocol analysis in Additional file 1: Table S3.

**Table 2:** Effect of intervention on primary outcome; disease control rate, medication management, and composite quality indicator

	**Baseline (T1)**		**Follow-up (T2)**		**Unadjusted**		**Fully adjusted**^**†**^	
	Control *n* (%)	Intervention *n* (%)	Control *n* (%)	Intervention *n* (%)	OR (95% CI)	*p* value	OR (95% CI)	*p* value
***Primary outcomes***								
Hypertension control (ref. = not controlled)	203 (89.4)	207 (95.8)	170 (90.9)	160 (93.6)	0.50 (0.19–1.29)	*0.1552*	0.54 (0.20–1.43)	*0.2129*
Diabetes control (ref. = not controlled)	83 (61.0)	62 (55.4)	62 (52.5)	61 (67.0)	2.28 (1.27–4.09)	*0.0057*	2.61 (1.37–4.99)	*0.0035*
Discontinued PIM (≥ 1) (ref. = equal or not reduced)			26 (15.8)	48 (42.1)	3.89 (2.22–6.80)	< *0.0001*	4.65 (2.41–8.97)	< *0.0001*
Reduction of medication in patients with polypharmacy (≥ 10) (ref. = equal or not reduced)			56 (35.9)	32 (34.8)	0.95 (0.55–1.63)	*0.8593*	1.40 (0.75–2.60)	*0.2863*
Reducing medication (≥ 1) (ref. = equal or not reduced)			79 (38.8)	85 (40.9)	1.28 (0.87–1.89)	*0.2050*	1.98 (1.24–3.16)	0.0042
	Control mean (SD)	Intervention mean (SD)	Control mean (SD)	Intervention mean (SD)	Estimate (SE)	*p* value	Estimate (SE)	*p* value
Number of PIMs	1.3 (1.1)	0.9 (1.0)	1.4 (1.2)	0.7 (1.0)	− 0.3018 (0.0717)	< *0.0001*	− 0.2666 (0.0648)	< *0.0001*
Improvement of CQI	0.3 (0.2)	0.3 (0.2)	0.3 (0.1)	0.3 (0.1)	0.0343 (0.0328)	*0.2966*	0.0327 (0.0260)	*0.2094*

^†^Fully adjusted for age, sex, log-transformed length of stay, cognitive performance scale, activities of daily living hierarchy scale, body mass index, number of total medications, institutional identification number

*CQI* composite quality indicators, *PIM* potentially inappropriate medication, *SE* standard error, *SD* standard deviation

Secondary outcomes, such as cognitive function, physical function, and HRQoL, did not significantly differ between the intervention and control groups (Additional file 1: Table S4). During the follow-up period, 34 patients (intervention; 19, control; 15) had at least 1 episode of unplanned hospitalization, but there was no significant difference between the intervention and control groups (*p* = 0.3379).

## Discussion

In this study, we successfully collected and analyzed data using the Health-RESPECT platform. Patients showed a significant improvement in diabetes control rate and medication management. In particular, the number of PIMs decreased in patients managed with the Health-RESPECT platform. This is the first study to show the benefits of information sharing and collaboration between specialists and physicians or nurses working at LTCHs or NHs. Considering the complexity of patient care and the shortage of specialists in the LTC setting, this novel platform is useful because it enables data collection, analysis, and provision of an evidence-based algorithm for disease management and patient care. Additionally, during the study period, the Health-RESPECT platform securely managed the participants’ data and operated stably. No patient safety event occurred.

As people age, integrated healthcare systems are needed to manage complex diseases, functional decline, and end-of-life care of older patients. Information sharing is an essential component of integrated healthcare networks for older patients. In particular, it is important to establish a medical record sharing system for communication among healthcare providers including specialists, doctors, and nurses. For example, the Extension for Community Health Outcomes–Care Transitions (ECHO-CT) model could identify safety errors related to discharge communication, coordination errors, or medication issues through a multidisciplinary telehealth videoconference program [30, 31].

In this study, the hypertension and diabetes control rates were higher than those of the average Korean population [32–35]. We adopted a cut-off value for blood pressure and glucose level considering age and frailty status; thus, the overall hypertension and diabetes control rates were expected to be higher than those of the general population due to the less strict cut-off level for control. For example, the control rate of hypertension in our study (89.4% of the control group and 95.8% of the intervention group at baseline) is much higher than the general older population (around 60%) [34]. However, it is possible that hypertension or diabetes among older patients living in LTCHs or NHs are overtreated. The Health-RESPECT program focuses on preventing adverse outcomes related to treatment by monitoring hypoglycemia, dizziness, orthostatic hypotension, hypotension, acute kidney injury, and electrolyte imbalance. So, the hypertension control in the intervention group could be decreased because physicians could respond to the alarm sign sent from Health-RESPECT. Several previous studies found that a substantial number of patients living in LTC settings were candidates for de-prescribing, and they benefited from a reduction in medication dosage [36–38]. Participants’ blood pressure and blood glucose levels in the present study were lower than the recommended target. In particular, we previously reported that the blood pressure level was lower in patients with frailty or cognitive impairment [39]. Thus, further deintensification of treatment may be beneficial without increasing the risk of adverse outcomes.

Polypharmacy is a common problem in older patients and is associated with an increased use of PIMs. In our Health-RESPECT platform, we included an automatic drug review system based on individual patients’ data and current evidence to support doctors. We found significant differences between the intervention and control groups in terms of the number of PIMs or patients who discontinued one or more PIM. Unfortunately, we could not show that PIM use reduction was associated with beneficial clinical outcomes in study participants. However, PIM has detrimental effects on older patients’ health status, and the 3-month follow-up period was too short to observe any positive effect. Thus, additional long-term studies are required to investigate whether reducing PIM use has favorable effects on older patients in LTCH or NH.

The COVID-19 pandemic has accelerated the use of ICT to manage chronic diseases or medication in LTCHs or NHs, improve patient access to health care, and optimize patient health outcomes. Previous study has shown a positive impact on the management of diabetes, hypertension, and rheumatoid arthritis among chronic diseases, mainly when consultation and monitoring methods were used [9]. It was observed that the ICT improves the overall quality of management through process improvements, such as access to specialists and medical efficacy in LTC facilities. A previous review reported preliminary effectiveness for supporting geriatric, psychiatric, and palliative care consultations through telemedicine in NHs [40]. For stable diffusion and maintenance of ICT, further research is required to explore stakeholders’ opinions, as well as to determine the costs required to introduce and workflow changes incurred with its use.

This study has many strengths. This is the first study to use a novel platform to provide data and evidence-based algorithm for disease management in an LTC setting. Using the platform, we can collect a wide range of data like vital signs, laboratory data, and prescription information without additional effort. Furthermore, the results of this study support the importance of information sharing and standardized care plans for older patients in LTCH or NH. The concept of data sharing used in this study can be applied to other countries that have similar nationwide HIE systems. Finally, this study was conducted in accordance with the CONSORT guidelines, and the CONSORT checklist is provided in Additional file 1: Table S5.

This study also has limitations. More than half of the cases excluded from the PP analysis are due to protocol violations during the follow-up period. Due to the COVID-19 pandemic, some of the 3-month follow-up data (*n* = 141) could not be collected within the designated period. At the time, as LTC institutions were locked down, measurement had to be delayed by 2–3 months from the scheduled follow-up date. However, the primary analysis of our study is ITT analysis and missing participants due to delayed follow-up assessment were included in ITT analysis. Moreover, the delay is not due to participants' health-related factors, so the high rate of attrition in PP analysis has little influence on the effect of intervention in this study. The attrition rate of this study is 25.4%, which is similar to the assumed attrition rate when we designed this study. A high attrition rate could influence selection bias. Thus, further research designs should consider the unique characteristics of patients in long-term care facilities.

Considering the differences in patients’ characteristics and the intervention performed in NH and LTCH, our results may not apply to the general population of older patients. We cannot evaluate the effect of our intervention on heart failure management due to an insufficient number of patients with heart failure. Due to the small number of cases (35 patients, 6.3% of the study participants), the statistical model to identify the effect of the intervention could not be computed, and values could not be determined.

We used a cluster design and randomized institutions to avoid contamination of the intervention in the control group. Nevertheless, because random assignment was based on institutions rather than individuals, there are statistically significant differences between the control and intervention groups at baseline variables measured at the individual level. Especially, the number of medications used at baseline differed between the intervention and the control groups, given the differences in drug use patterns by LTC institutions. We statistically adjusted for the variables with significant differences in the final analytical models.

Furthermore, the lack of blinding could be considered a limitation. Physicians and healthcare professionals in the control group were aware of the trial’s purpose, which might have influenced their clinical practice more than when providing usual care. Lastly, previous studies reported that technology-led multidisciplinary care can improve the quality of life—as assessed by health and functional outcomes—in frail older people residing in NHs; however, in our study, only some of the outcomes showed improvement [26]. The limited results of the present study might be related to the greater medical needs of patients in LTCHs rather than NHs and the short intervention period. Moreover, the saturated blood pressure control rate at baseline makes it difficult to infer the effectiveness of the Health-RESPECT [37].

Until now, telephone consultation tended to be a dominant mode of telehealth service delivery in LTC facilities. The huddle to introduce ICT may differ by the reimbursement system, electronic prescription system, and workforce. The utilization of ICT in healthcare may be restricted due to various factors such as patients' and healthcare professionals' perceptions, technical difficulties and equipment-related problems, and inadequate knowledge of ICT usage among healthcare workers. Sensory or cognitive impairment in older patients may also pose additional challenges [9]. Owing to the COVID-19 pandemic, telehealth has continued to be an alternative platform for providing clinical care. However, a healthcare platform that introduces ICT with improved clinical efficiency, has a user-friendly interface, ensures appropriate compensation, is based on evidence from various medical fields, and caters to the changing traditional mindset is essential to shift the paradigm to ICT use in LTCHs and NHs.

## Conclusions

The ICT-supported integrated health service was effective in reducing PIM use and improving diabetes control among older patients staying in LTCH or NH without a functional decline or increased healthcare utilization. Considering the burden of care for older patients in the LTC setting, more collaborative systems between acute care and LTCH or NH must be developed.

### Supplementary Information

Additional file 1: Figure S1. Component and architecture of the Health-RESPECT platform designed for information exchange between the long-term care setting and referral hospital. Figure S2. Algorithm for disease management incorporated in the Health-RESPECT platform; (A) Hypertension management, (B) Diabetes mellitus management, (C) Heart failure management. Table S1. List of potentially inappropriate medications. The list of potentially inappropriate medications was developed based on Beers Criteria and considering the medical environment of the long-term care facilities in Korea. Table S2. Outcome variables, definitions and timeline. Table S3. Effect of intervention on secondary outcomes (the intention to treat analysis). Table S4. Effect of intervention on primary outcome (the per protocol analysis). Table S5. CONSORT 2010 checklist of information to include when reporting a randomised trial.

## Data Availability

Data used during the current study are available from the corresponding author upon reasonable request.

## Abbreviations

ADL: Activities of daily living
BMI: Body mass index
COVID-19: The coronavirus disease 2019
CQI: Composite quality indicator
EQ-5D: EuroQol 5-dimensions
EQ-VAS: EuroQol visual analog scale
FAC: Functional ambulation category
GLMM: Generalized linear mixed effects model
Health-RESPECT: Integrated caRE Systems for elderly PatiEnts using iCT
HIE: Health information exchange
HRQoL: Health-related quality of life
ICT: Information and communication technology
ITT: Intention-to-treat
K-FRAIL: Korean version of the FRAIL
LMM: Linear mixed effects model
LTCHs: Long-term care hospitals
MMSE-K: Mini-Mental State Examination
NHs: Nursing homes
OR: Odds ratio
PIM: Potentially inappropriate medication
PP: Per protocol
