# Supplementary material for: Information technology-supported integrated health service for older adults in long-term care settings
Source: BMC Med. 2024 May 29;22:212. doi: 10.1186/s12916-024-03427-7 (PMC11134747; doi:10.1186/s12916-024-03427-7)
Supplement: Supplementary file 1 — Additional file 1: Figure S1. Component and architecture of the Health-RESPECT platform designed for information exchange between the long-term care setting and referral hospital. Figure S2. Algorithm for disease management incorporated in the Health-RESPECT platform; (A) Hypertension management, (B) Diabetes mellitus management, (C) Heart failure management. Table S1. List of potentially inappropriate medications. The list of potentially inappropriate medications was developed based on Beers Criteria and considering the medical environment of the long-term care facilities in Korea. Table S2. Outcome variables, definitions and timeline. Table S3. Effect of intervention on secondary outcomes (the intention to treat analysis). Table S4. Effect of intervention on primary outcome (the per protocol analysis). Table S5. CONSORT 2010 checklist of information to include when reporting a randomised trial. [file 12916_2024_3427_MOESM1_ESM.docx]

**Figure S1. Component and architecture of the Health-RESPECT (integrated caRE Systems for elderly PatiEnts using iCT) platform designed for information exchange between the long-term care setting and referral hospital**


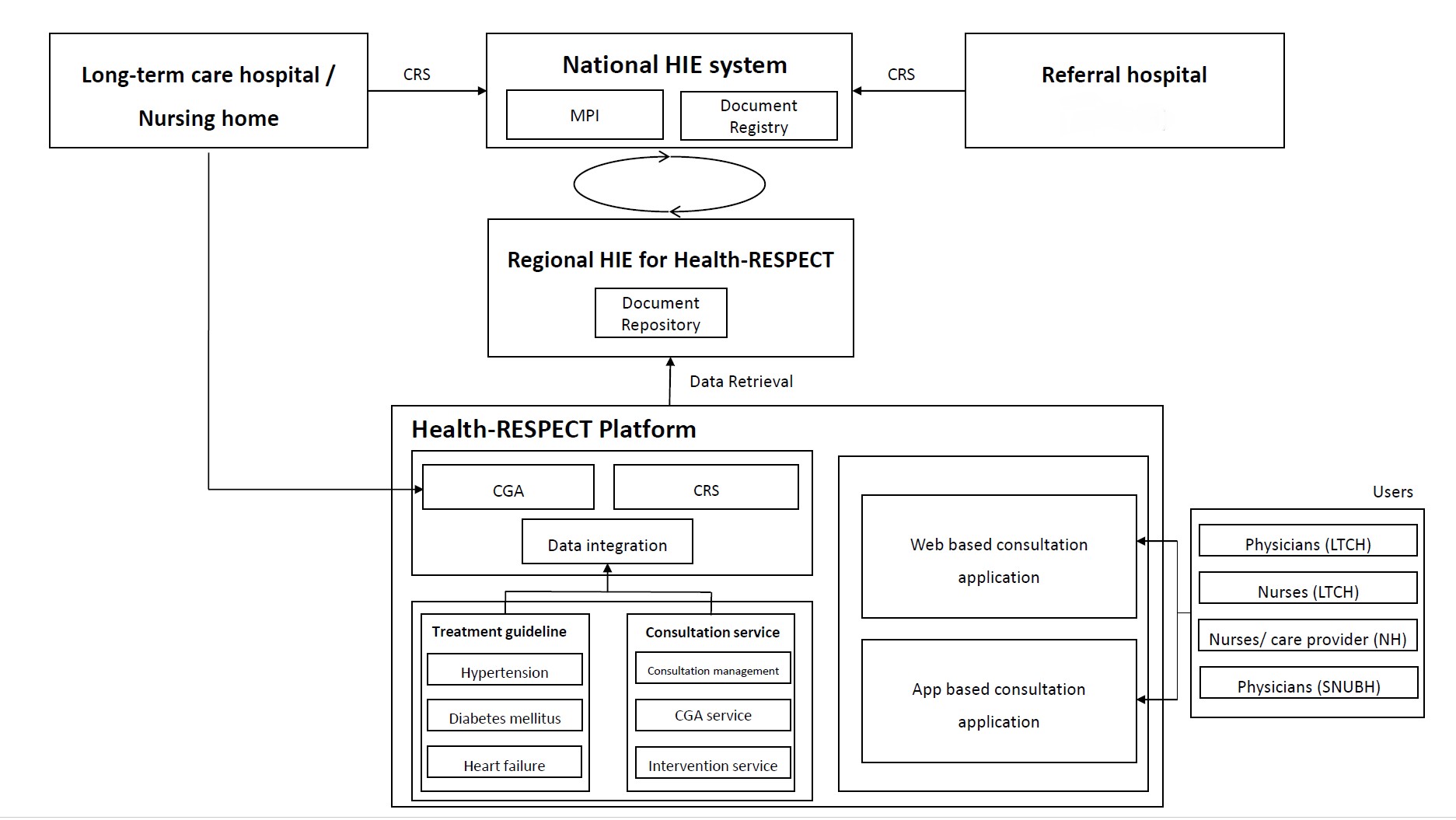


CGA, comprehensive geriatric assessment; CRS, care record summary; HIE, health information exchange; LTCH, long-term care hospital; MPI, master patient index; NH, nursing home

**Figure S2. Algorithm for disease management incorporated in the Health-RESPECT platform; (A) Hypertension management, (B) Diabetes mellitus management, (C) Heart failure management**


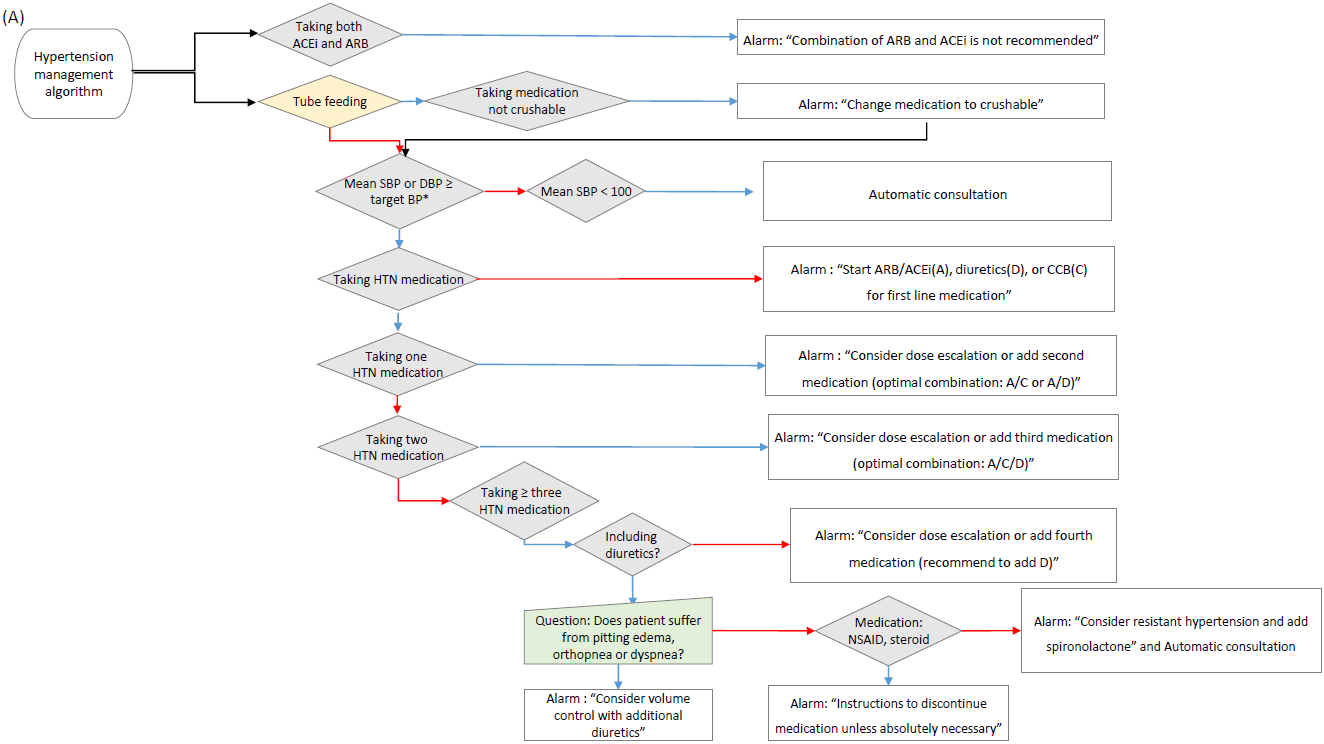


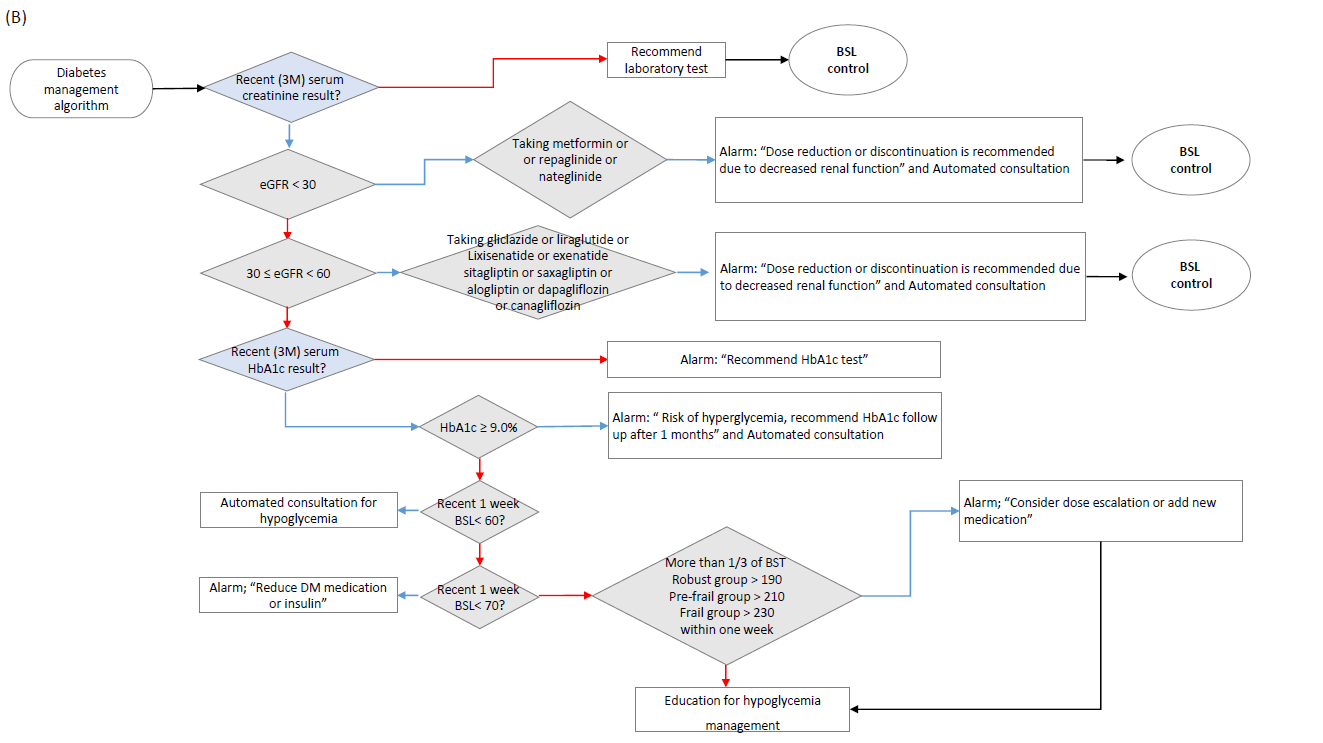


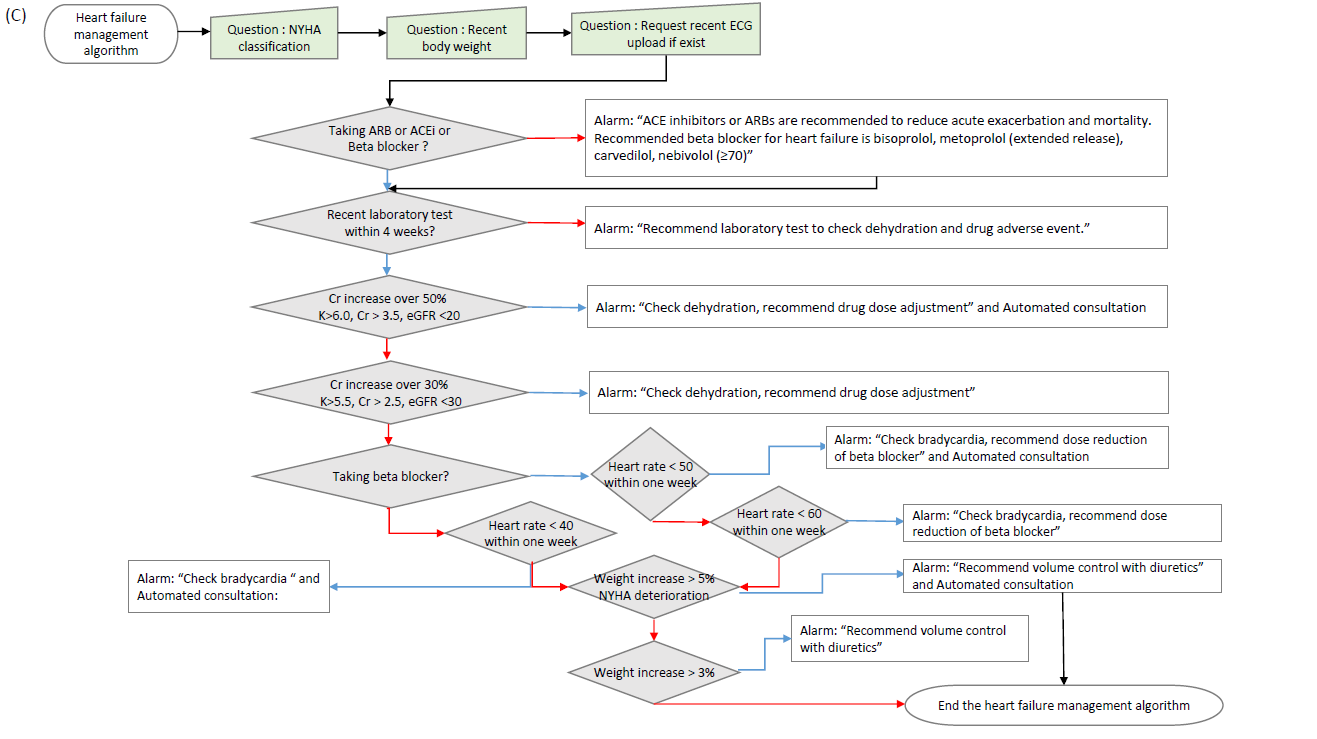


ACEi, angiotensin converting enzyme inhibitor; ARB, angiotensin receptor blocker; BSL, blood sugar level; DBP, diastolic blood pressure; eGFR, estimated glomerular filtration rate; HTN, hypertension; NSAID, non-steroidal anti-inflammatory drug; NYHA, New York Heart Association; SBP, systolic blood pressure

**Table S1. List of potentially inappropriate medications**

The list of potentially inappropriate medications was developed based on Beers Criteria and considering the medical environment of the long term care facilities in Korea

| **Organ system/**  **Therapeutic category** | **Medications** |
| --- | --- |
| **Anticholinergics** | **First-generation antihistamines**  - Chlorpheniramine  - Clemastine  - Cyproheptadine  - Cinnarizine  - Diphenhydramine  - Hydroxyzine  - Triprolidine |
|  | **Antiparkinson agents**  - Benztropine  - Trihexyphenidyl |
|  | **Antispasmodics**  - Atropine  - Belladonnaalkaloids  - Clidinium-Chlordiazepoxide  - Scopolamine |
| **Antithrombotics** | - Dipyridamole, oral short acting  - Ticlopidine |
| **Cardiovascular** | **Peripheral alpha-1 blockers**  - Doxazosin  - Prazosin  - Terazosin |
|  | **Anti-arrhythmic agents**  - Amiodarone  - Dronedarone  - Digoxin |
|  | **Central alpha blockers**  - Clonidine  - Methyldopa  - Moxonidine |
|  | **Caclium channel blocker**  - Short acting nifedipine |
| **Central Nervous** | **Antidepressants**  - Amitriptyline  - Amoxapine  - Clomipramine  - Doxepin>6mg/day  - Imipramine  - Nortriptyline  - Paroxetine |
|  | **Barbiturates**  - Phenobarbital  - Pentobarbital |
|  | **Antipsychotics**  **1^st^(conventional)generation**  - Sulpiride  - Chlorpromazine  **2^nd^(atypical)generation**  - Amisulpride,  - Ziprasidone  - Clozapine  - Olanzapine  - Aripiprazole  - Zotepine  - Paliperidone  **Lithium** |
|  | **Benzodiazepines**  **Short and Intermediate acting**  - Alprazolam  - Clotiazepam  - Etizolam  - Lorazepam  - Triazolam  - Tofisopam  **Long acting**  - Chlordiazepoxide  - Clonazepam  - Diazepam  - Clobazam  - Pinazepam  - Flurazepam |
|  | **Nonbenzodiazepine, hypnotics**  -Zolpidem |
|  | **Ergoloid mesylates** |
| **Genitourinary** | Desmopressin |
| **Endocrine** | Megestrol |
| **Gastrointestinal** | - Metoclopramide  - Levosulpiride |
|  | **Proton-pump inhibitors(PPIs)**  - Esomeprazole  - Lansoprazole  - Omeprazole  - Pantoprazole  - Rabeprazole |
| **Pain** | Meperidine |
|  | **NSAID**  - Indomethacin  - Ketorolac,includes parenteral |
|  | **Non–COX-selective NSAIDs, oral**  - Aceclofenac  - Dexibuprofen  - Diclofenac  - Dexketoprofen  - Fenoprofen  - Ibuprofen  - Ketoprofen  - Meloxicam  - Nabumetone  - Naproxen  - Piroxicam  - Sulindac |
|  | **Skeletal muscle relaxants**  - Chlorzoxazone  - Cyclobenzaprine  - Methocarbamol  - Orphenadrine |
|  | **Systemic Steroid**  - Methylprednisolone  - Prednisolone  - Triamcinolone |

**Table S2 Outcome variables, definition and timeline.**

| **Variables** | **Definition** | **Timeline** | |
| --- | --- | --- | --- |
|  |  | **T1** | **T2** |
| **Primary outcomes** | | | |
| Hypertension control | Target blood pressure for hypertension is 140/90 mm Hg in the robust and prefrail groups and 150/90 mm Hg in frail groups.* | X | X |
| Diabetes control | The target Hemoglobin A1c (HbA1c) for diabetes is <7.5% in robust groups, <8.0% in prefrail groups, and <8.5% in frail groups, or for random glucose level, the target is ≤190 mg/dL in robust groups, ≤210 mg/dL in prefrail groups and ≤230 mg/dL in frail groups. * | X | X |
| Inappropriate medications | - the number of potentially inappropriate medications (PIMs)  - discontinuation of PIM | X | X |
| Overall functional status with a composite indicator | A series of care quality indicators based on functional status in the interRAI Long-term Care Facilities (LTCF) Tool | X | X |
| **Secondary outcomes** | | | |
| Functional rehabilitation management | Cognitive function: Korean Mini-Mental State Examination  Motor function: Functional Ambulation Category  Swallowing problem: pneumonia incidence | X | X |
| Functional status with individual indicators | Individual care quality indicators based on functional status in the interRAI Long-term Care Facilities (LTCF) Tool | X | X |
| Quality of life | EuroQol- 5 Dimension Korean version | X | X |
| Acute healthcare utilization | Hospitalization through the emergency room and emergency room visit | X | X |
| Patient and healthcare professional experience | Focused group discussion |  | X |
| Technology acceptance | - Brooke’s System Usability Scale  - Telehealth Usability Questionnaire tool |  | X |
| Cost-effectiveness | Incremental cost-effectiveness ratio.  (Medical costs, non-medical costs, and program costs are included in the cost, and the index of primary outcomes is used for effectiveness.) | X | X |
| Willingness to pay | For the cost–benefit analysis, the benefit is measured by willingness to pay. To measure the subjective utility of healthcare, willingness to pay is measured for healthcare personnel. Net benefits are calculated by deducting the total cost from the willingness to pay values |  | X |

*Frailty status was evaluated with the Korean version of the FRAIL (Fatigue, Resistance, Ambulation, Illness, and Loss of weight, K-FRAIL) scale. Scores of 3 and more, 1–2 and 0, were classified as frail, prefrail, and robust, respectively.

**Table S3. Effect of intervention on primary outcome (the per protocol analysis)**

|  | **Baseline (T1)** | | **Follow up (T2)** | | **Unadjusted** | | **Fully adjusted^†^** | |
| --- | --- | --- | --- | --- | --- | --- | --- | --- |
|  | Control group n (%) | Intervention group n (%) | Control group n (%) | Intervention group n (%) | OR (95% CI) | p value | OR (95% CI) | p value |
| ***Primary analysis*** |  |  |  |  |  |  |  |  |
| *Hypertension control | 129 (86.6) | 104 (96.3) | 134 (89.9) | 103 (92.8) | 0.35 (0.11 - 1.15) | 0.0834 | 0.33 (0.10 - 1.06) | 0.0630 |
| *Diabetes control | 63 (60.0) | 35 (60.3) | 57 (52.8) | 37 (64.9) | 1.69 (0.84 - 3.42) | 0.1435 | 2.00 (0.96 - 4.20) | 0.0660 |
| ^+^Discontinued PIM |  |  | 23 (16.6) | 36 (36.6) | 2.91 (1.51 - 5.63) | 0.0014 | 3.41 (1.59 - 7.32) | 0.0016 |
| ^+^Reduction of medication in patients with polypharmacy (≥10) |  |  | 54 (36.5) | 21 (33.9) | 0.89 (0.48 - 1.66) | 0.7183 | 3.41 (1.59 - 7.32) | 0.0016 |
| ^+^Reducing medication |  |  | 75 (39.1) | 47 (37.9) | 0.94 (0.59 - 1.50) | 0.8082 | 1.44 (0.83 - 2.48) | 0.1971 |
|  | Control group mean (SD) | Intervention group mean (SD) | Control group mean (SD) | Intervention group mean (SD) | Estimate (SE) | p value | Estimate (SE) | p value |
| Number of PIMs | 1.4 (1.2) | 0.9 (1.0) | 1.5 (1.3) | 0.7 (1.0) | -0.2850 (0.0840) | 0.0008 | -0.2944 (0.0744) | <.0001 |
| Improvement of CQI | 0.3 (0.2) | 0.4 (0.2) | 0.3 (0.2) | 0.3 (0.1) | -0.0039 (0.0498) | 0.9373 | 0.0183 (0.0398) | 0.6463 |

*Reference was not controlled, ^+^reference was equal or not reduced number of PIM or medications ^†^Fully adjusted for age, sex, log-transformed length of stay, cognitive performance scale, activities of daily living hierarchy scale, body mass index, number of total medications, institutional identification number

CQI, composite quality indicators; PIM, potentially inappropriate medication; PP, per protocol; SE, standard error; SD, standard deviation

**Table S4.** **Effect of intervention on secondary outcomes (the intention to treat analysis)**

|  | **Baseline (T1)** | | **Follow up (T2)** | | **Unadjusted** | | **Fully adjusted^†^** | |
| --- | --- | --- | --- | --- | --- | --- | --- | --- |
|  | Control group  mean (SD) | Intervention group  mean (SD) | Control group  mean (SD) | Intervention group  mean (SD) | Estimate  (SE) | p value | Estimate (SE) | p value |
| ***Cognitive functions*** |  |  |  |  |  |  |  |  |
| MMSE | 11.4 (8.7) | 13.3 (8.0) | 11.2 (9.0) | 12.8 (8.7) | 0.2531  (0.3597) | 0.4822 | 0.5179  (0.3938) | 0.1892 |
| ***Physical functions*** |  |  |  |  |  |  |  |  |
| FAC | 0.8 (1.3) | 0.9 (1.4) | 0.8 (1.3) | 1.0 (1.5) | 0.0012  (0.0727) | 0.9869 | -0.0124 (0.0765) | 0.8715 |
| ***Quality of life*** |  |  |  |  |  |  |  |  |
| EQ-5D | 0.55 (0.26) | 0.51 (0.27) | 0.55 (0.26) | 0.53 (0.28) | 0.0274  (0.0208) | 0.1873 | 0.0235  (0.0235) | 0.2646 |

EQ-5D, EuroQol 5-Dimensions utility weight; FAC, functional ambulation categories; ITT, intention to treat; MMSE, mini-mental state examination; SE, standard error; SD, standard deviation

^†^Fully adjusted for age, sex, log-transformed length of stay, cognitive performance scale, activities of daily living hierarchy scale, body mass index, number of total medications, institutional identification number

Table S5.
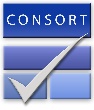
 CONSORT 2010 checklist of information to include when reporting a randomised trial

| Section/Topic | No | Checklist item | page No |
| --- | --- | --- | --- |
| Title and abstract | | | |
|  | 1a | Identification as a randomised trial in the title | 21 |
|  | 1b | Structured summary of trial design, methods, results, and conclusions (for specific guidance see CONSORT for abstracts) | 3-4 |
| Introduction | | | |
| Background and objectives | 2a | Scientific background and explanation of rationale | 4-5 |
|  | 2b | Specific objectives or hypotheses | 5 |
| Methods | | | |
| Trial design | 3a | Description of trial design (such as parallel, factorial) including allocation ratio | 6 |
|  | 3b | Important changes to methods after trial commencement (such as eligibility criteria), with reasons | NA |
| Participants | 4a | Eligibility criteria for participants | 6 |
|  | 4b | Settings and locations where the data were collected | 8-9 |
| Interventions | 5 | The interventions for each group with sufficient details to allow replication, including how and when they were actually administered | 8 |
| Outcomes | 6a | Completely defined pre-specified primary and secondary outcome measures, including how and when they were assessed | 9, Supplementary |
|  | 6b | Any changes to trial outcomes after the trial commenced, with reasons | NA |
| Sample size | 7a | How sample size was determined | 10 |
|  | 7b | When applicable, explanation of any interim analyses and stopping guidelines | NA |
| Randomisation: |  |  |  |
| Sequence generation | 8a | Method used to generate the random allocation sequence | NA |
|  | 8b | Type of randomisation; details of any restriction (such as blocking and block size) | 6, 10 |
| Allocation concealment mechanism | 9 | Mechanism used to implement the random allocation sequence (such as sequentially numbered containers), describing any steps taken to conceal the sequence until interventions were assigned | 6, 10 |
| Implementation | 10 | Who generated the random allocation sequence, who enrolled participants, and who assigned participants to interventions | NA |
| Blinding | 11a | If done, who was blinded after assignment to interventions (for example, participants, care providers, those assessing outcomes) and how | 9 |
|  | 11b | If relevant, description of the similarity of interventions | NA |
| Statistical methods | 12a | Statistical methods used to compare groups for primary and secondary outcomes | 10 |
|  | 12b | Methods for additional analyses, such as subgroup analyses and adjusted analyses | 10 |
| Results | | | |
| Participant flow (a diagram is strongly recommended) | 13a | For each group, the numbers of participants who were randomly assigned, received intended treatment, and were analysed for the primary outcome | 11 |
|  | 13b | For each group, losses and exclusions after randomisation, together with reasons | 11, Figure 2 |
| Recruitment | 14a | Dates defining the periods of recruitment and follow-up | 6 |
|  | 14b | Why the trial ended or was stopped | NA |
| Baseline data | 15 | A table showing baseline demographic and clinical characteristics for each group | 8-7 |
| Numbers analysed | 16 | For each group, number of participants (denominator) included in each analysis and whether the analysis was by original assigned groups | 8 |
| Outcomes and estimation | 17a | For each primary and secondary outcome, results for each group, and the estimated effect size and its precision (such as 95% confidence interval) | 8,10-11 |
|  | 17b | For binary outcomes, presentation of both absolute and relative effect sizes is recommended | 3 |
| Ancillary analyses | 18 | Results of any other analyses performed, including subgroup analyses and adjusted analyses, distinguishing pre-specified from exploratory | NA |
| Harms | 19 | All important harms or unintended effects in each group (for specific guidance see CONSORT for harms) | 16 |
| Discussion | | | |
| Limitations | 20 | Trial limitations, addressing sources of potential bias, imprecision, and, if relevant, multiplicity of analyses | 18-19 |
| Generalisability | 21 | Generalisability (external validity, applicability) of the trial findings | 18 |
| Interpretation | 22 | Interpretation consistent with results, balancing benefits and harms, and considering other relevant evidence | 16-19 |
| Other information | | |  |
| Registration | 23 | Registration number and name of trial registry | 3 |
| Protocol | 24 | Where the full trial protocol can be accessed, if available | Reference 11, 12 |
| Funding | 25 | Sources of funding and other support (such as supply of drugs), role of funders | 21 |
